# High-Moisture Extrusion of a Dietary Protein Blend Impairs In Vitro Digestion and Delays In Vivo Postprandial Plasma Amino Acid Availability in Humans

**DOI:** 10.1016/j.tjnut.2024.05.018

**Published:** 2024-05-24

**Authors:** Sam West, Alistair J Monteyne, Gráinne Whelehan, Doaa R Abdelrahman, Andrew J Murton, Tim JA Finnigan, Giuseppina Mandalari, Catherine Booth, Peter J Wilde, Francis B Stephens, Benjamin T Wall

**Affiliations:** 1 Department of Public Health and Sport Sciences, Faculty of Health and Life Sciences, University of Exeter, Exeter, United Kingdom; 2 Nuffield Department of Primary Care Health Sciences, University of Oxford, Oxford, United Kingdom; 3 NIHR Oxford Biomedical Research Centre, Oxford, University Hospitals NHS Foundation Trust, Oxford, United Kingdom; 4 Department of Surgery, University of Texas Medical Branch, Galveston, TX, United States; 5 Sealy Center on Aging, University of Texas Medical Branch, Galveston, TX, United States; 6 New Era Foods, Hutton Rudby, Yarm, United Kingdom; 7 Department of Chemical, Biological, Pharmaceutical and Environmental Science, University of Messina, Messina, Italy; 8 Quadram Institute Bioscience, Norwich Research Park, Norwich, Norfolk, United Kingdom

**Keywords:** mycoprotein, pea protein, bioavailability, digestion, amino acids, extrusion, INFOGEST

## Abstract

**Background:**

Industrial processing can alter the structural complexity of dietary proteins and, potentially, their digestion and absorption upon ingestion. High-moisture extrusion (HME), a common processing method used to produce meat alternative products, affects in vitro digestion, but human data are lacking. We hypothesized that HME of a mycoprotein/pea protein blend would impair in vitro digestion and in vivo postprandial plasma amino acid availability.

**Methods:**

In Study A, 9 healthy volunteers completed 2 experimental trials in a randomized, double-blind, crossover design. Participants consumed a beverage containing 25 g protein from a “dry” blend (CON) of mycoprotein/pea protein (39%/61%) or an HME content-matched blend (EXT). Arterialized venous blood samples were collected in the postabsorptive state and regularly over a 5-h postprandial period to assess plasma amino acid concentrations. In Study B, in vitro digestibility of the 2 beverages were assessed using bicinchoninic acid assay and optical fluorescence microscopy at baseline and during and following gastric and intestinal digestion using the INFOGEST model of digestion.

**Results:**

Protein ingestion increased plasma total, essential (EAA), and branched-chain amino acid (BCAA) concentrations (time effect, *P* < 0.0001) but more rapidly and to a greater magnitude in the CON compared with the EXT condition (condition × time interaction, *P* < 0.0001). This resulted in greater plasma availability of EAA and BCAA concentrations during the early postprandial period (0–150 min). These data were corroborated by the in vitro approach, which showed greater protein availability in the CON (2150 ± 129 mg/mL) compared with the EXT (590 ± 41 mg/mL) condition during the gastric phase. Fluorescence microscopy revealed clear structural differences between the 2 conditions.

**Conclusions:**

These data demonstrate that HME delays in vivo plasma amino acid availability following ingestion of a mycoprotein/pea protein blend. This is likely due to impaired gastric phase digestion as a result of HME-induced aggregate formation in the pea protein.

This trial was registered at clinicaltrials.gov as NCT05584358.

## Introduction

Dietary protein quality is largely determined by the amino acid composition of a protein source and the digestibility of that protein [[1], [2], [3]]. In turn, these 2 factors dictate the postprandial plasma availability (rapidity, magnitude, and total bioavailability) and consequent metabolic fate of the ingested amino acids [4,5]. For example, a wealth of research now demonstrates that the amino acid content [1,6], bioavailability [7], and the resultant increase in muscle protein synthesis [4,8] all vary depending on the protein source ingested. However, protein *form* (that is, the variation in which we ingest the *same* source of protein) can also influence the postprandial appearance of amino acids in circulation. For instance, ingesting protein as whole foods compared with isolated versions [9,10], different textures [11], following various methods of cooking [[12], [13], [14]], heat treatment [15], and hydrolysis [16] can all modulate postprandial amino acid availability. Dietary protein form can also be modulated by various industrial processing techniques [17,18]. Such processes are often overlooked within the investigation of dietary protein metabolism but are of clear relevance given that most proteins available for public consumption have been subjected to industrial processing of some description [19].

High-moisture extrusion (HME) is a method commonly used to produce meat replacement products and is a promising future food technology [[20], [21], [22]]. HME is a thermomechanical process in which components undergo complex changes in temperature, pressure, and shear resulting in physical and chemical changes to allow blending and texturization of different protein sources [23]. Although this process is commonly used commercially to impart texture in meat replacement products, the physicochemical changes altering the structural complexity of the food and how this will impact human digestion are poorly understood [24]. However, in vitro investigations into the digestion of extruded proteins have observed both improved [[25], [26], [27]] and impaired [[28], [29], [30]] digestion, with the disparity likely explained by the variety of protein sources studied [31] and/or specific HME parameters [32].

Assessing how the structural complexity of a protein source modulates protein digestibility in humans is subject to methodological constraints. Oro-ileal assays and intrinsically labeled protein methods can provide insight into the “true” protein digestibility and amino acid bioavailability, respectively [33,34]. However, they are complex and/or resource-intensive to conduct and do not allow for the assessment of the behavior of protein throughout the gastrointestinal (GI) tract. The INFOGEST static method of digestion is a standardized, validated in vitro method used to simulate the environment of human digestion [35]. The frequent sampling throughout the different stages of digestion permits the assessment of protein hydrolysis, availability, and structure, presenting mechanistic insights into the behavior of a protein source throughout digestion.

Pea protein (PP) isolate and mycoprotein (MYC) are sustainably produced non–animal-derived protein sources [36,37] with demonstrated utility in human nutrition [10,38]. However, there are currently no data on the effects of HME on MYC, and in vitro assessments of PP following HME show an impairment in digestibility due to heat-induced aggregate formation [29,30]. In the present study, we tested the hypothesis that HME would impair digestibility and postprandial plasma amino acid availability of a MYC/PP blend. We tested this hypothesis with a multidisciplinary approach comprising 2 separate experiments: Study A: in an in vivo human volunteer experiment, we compared plasma amino acid concentrations following bolus ingestion of a MYC/PP (39%/61%) dry blend or an identical blend that had undergone HME; Study B: using the INFOGEST static model of digestion, we assessed in vitro protein digestion and subsequent bioaccessibility of amino acids of the same dry and HME protein blends.

## Methods

### Study A

#### Participants and medical screening

Nine young healthy individuals volunteered to participate in the present study (age: 21 ± 1 y; body mass: 73 ± 4 kg; BMI: 24 ± 1 kg/m^2^; 5 male, 4 female). Participants’ characteristics are shown in Table 1. Before inclusion in the study, all participants completed a routine screening to assess eligibility to participate and during which measures of height, weight, BMI, resting blood pressure, and body composition were assessed. Body composition (body fat [percentage] and lean mass [kilograms]) was assessed using air displacement plethysmography (BodPod, Life measurement, Inc.). Subjects were admitted to the study after being deemed healthy based on blood pressure (<140/90 mmHg), BMI (18–30 kg/m^2^), and responses to a routine medical health questionnaire. Exclusion criteria included any diagnosed metabolic or digestive impairment, cardiovascular complications, or allergies to MYC/Quorn/edible fungi, penicillin, or environmental molds. Experimental procedures, potential risks, and the purpose of the study were explained to the participants prior to obtaining informed written consent. This study was approved by the Sport and Health Sciences ethics committee of the University of Exeter (21-10-20-A-06) in accordance with the Declaration of Helsinki and is registered at clinicaltrials.gov (NCT05584358). Recruitment and data collection were carried out in the Nutritional Physiology Research Unit at the University of Exeter between February 2022 and August 2022.

**TABLE 1:** Participants’ characteristics

	Mean ± SEM (*n* = 9)
Sex (M/F)	5/4
Age, y	21 ± 1
Height, cm	174 ± 4
Weight, kg	73 ± 4
BMI, kg/m^2^	24 ± 1
Systolic blood pressure (mmHg)	122 ± 3
Diastolic blood pressure (mmHg)	68 ± 2
Fat, % body mass	22 ± 3
Lean mass, kg	57 ± 4

#### Experimental design

**FIGURE 1:**
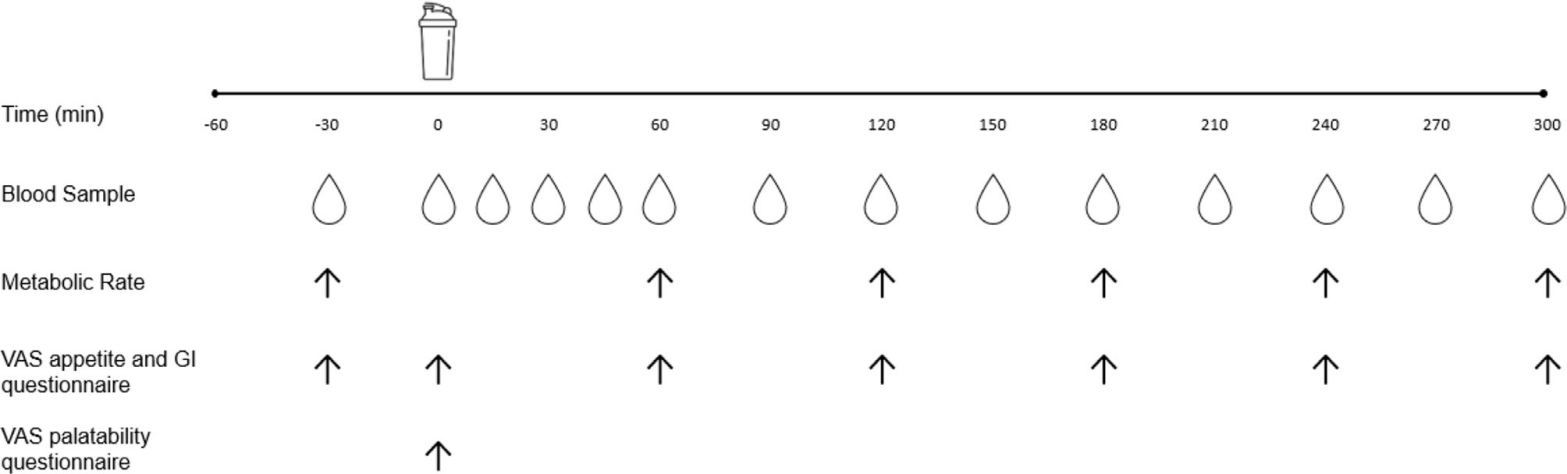
Protocol schematic of the experimental visit. GI, gastrointestinal; VAS, visual analog scale.

A schematic overview of the protocol used in Study A can be found in Figure 1. In a randomized, double-blind, crossover design, participants completed 2 laboratory test days (Supplementary Figure 1). During each visit, participants ingested a beverage containing 25 g of protein from either a dry milled MYC/PP blend (CON) or an extruded MYC/PP blend (EXT). Volunteers attended the laboratory for each visit (counterbalanced for order) separated by a minimum of 5 d to ensure complete digestion, absorption, and metabolism of the test beverages. Participants were asked to refrain from strenuous physical activity and alcohol consumption 48 h before each visit. All participants were provided with a standardized meal (4.6 MJ [1110 kcal], 29% energy from fat, 46% energy from carbohydrate, 25% energy from protein) to consume as their last food intake ∼10 h before arriving at the laboratory.

Participants arrived at the laboratory at 08:00 in the fasted state, voided their bladder, and rested in a semisupine position for 30 min. Thereafter, resting total energy expenditure (TEE) and substrate oxidation rates were assessed using expired gas collections for indirect calorimetry via a mixing chamber that obtained 20-s averages (Cortex Metalyzer 2R gas analyzer). Expired gases were collected to obtain $\dot{V}O_{2}$ and $\dot{V}{\text{CO}}_{2}$ for determination of substrate oxidation rates according to nonprotein stoichiometric equations [39]. TEE during this period was calculated as the sum of energy production from fat and carbohydrate, assuming that the oxidation of 1 g of triacylglyceride (862 g/mol) liberates 39.4 kJ and 1 g of glucose (180 g/mol) liberates 15.6 kJ of energy. This baseline measurement was collected for 20 min with data accepted as the average between 5 and 15 min [40]. Participants then completed subjective appetite visual analog scales (VASs). These 100-mm paper-based scales detailed questions regarding fullness, hunger, satisfaction, prospective food consumption, and satiety, which were anchored by diametrically opposed feelings of extremity [41]. Volunteers reported their perceived feelings on each scale in the same order each time. Ratings were subsequently measured by the same researcher each time to minimize discrepancies and used to calculate an appetite score [41]. Thereafter, a cannula was inserted retrogradely into a dorsal hand vein, and the hand was placed in a heated hand unit (55°C) to allow for subsequent arterialized venous blood sampling [42]. The hand was kept in the heated hand unit for 15 min to allow for arterialization of the venous blood flow, after which a fasting blood sample was collected. Participants then ingested the test beverage, initiating a 5-h postprandial period. Participants were instructed to consume the beverage in under 5 min, and the precise time was noted and matched in the second visit. Arterialized venous blood samples were collected at 15, 30, 45, 60, 90, 120, 150, 180, 240, 270, and 300 min following drink ingestion. Further indirect calorimetry measurements were collected for the final 15 min of each hour (i.e., collection of measurement completed at 60, 120, 180, 240, and 300 min into the postprandial period) following beverage ingestion. Participants completed VASs at 5, 60, 120, 180, 240, and 300 min after drink consumption. The final blood sample signified the end of the experiment after which the cannula was removed, and participants were provided with food before leaving the laboratory.

#### Protein beverage preparation

Both protein blends were provided by Marlow Foods Ltd, Quorn Foods. Natural and freeze-dried MYC was produced by Marlow Foods, as previously described [37]. Freeze-dried PP was produced and supplied by Corsucra Ltd. The dry blend (CON) was produced by mixing the freeze-dried powders (39% MYC, 61% PP). The extrudate was produced using a Buhler twin-screw extruder (Buhler group). The length of the extruder barrel was 2533 mm. The barrel contained 10 zones and a cooling die (Buhler Poly-Cool 500). The temperatures of the zones were as follows; zone 1, 90°C; zones 2–3, 90°C; zones 4–6, 160°C; zones 7–8, 168°C; zones 9–10, 111°C; cooling die, 84°C, with a constant pressure of 16 bar. The screw speed was 500 × *g*. MYC, PP isolate, and water were simultaneously fed into the extruder at 104, 147, and 79 kg/h, respectively (39% MYC, 61% PP). The viscous mix was then collected from the end of the cooling die, cooled, and freeze-dried to produce a protein powder. Both blends were independently analyzed (Premier Analytical Services) for energy and macronutrient content and amino acid composition, the details of which are displayed in Table 2.

**TABLE 2:** The nutritional composition of the experimental beverages (31.9 g and 31.4 g of CON and EXT, respectively)

	CON	EXT
Macronutrients		
Protein, g	25	25
Carbohydrate, g	1	1
Fat, g	2	2
Fiber, g	0	2
Energy, kcal	125	125
Energy, kJ	529	530
Amino acid content, g		
Alanine	1.2	1.2
Arginine	2.1	2.1
Asparagine	3.1	2.7
Cysteine	0.2	0.5
Glutamine	4.4	4.0
Glycine	1.1	1.1
Histidine	0.6	0.6
Isoleucine	0.9	1.2
Leucine	1.9	1.8
Lysine	2.0	1.8
Methionine	0.3	0.3
Phenylalanine	1.3	1.4
Proline	1.1	1.2
Serine	1.5	1.4
Threonine	0.9	1.0
Tryptophan	0.2	0.2
Tyrosine	0.9	0.9
Valine	1.0	1.6

Abbreviations: CON, dry blend; EXT, extruded blend.

Protein content (g) is calculated from the sum of the amino acids measured after complete hydrolysis.

The evening before each visit, the powdered protein sources were assimilated with 350 mL water and 40 mL energy-free flavoring (Clearwater), blended for ∼2 min, and refrigerated overnight. During the experimental trial, once participants had consumed the drink, an additional 100 mL water was added to rinse the bottle and ensure that all the protein had been consumed, leading to a total fluid volume consumed of 490 mL. Double-blinding of the drinks was achieved by having a separate researcher from those carrying out the experimental trial visits prepare the drinks in an opaque bottle. Following beverage consumption, participants were asked to identify the protein beverage they consumed, with 38% (7 out of 18) correct guesses, implying success of the blinding procedures. The drinks were matched for protein content (25 g) requiring 31.9 g and 31.4 g of the dry and extruded blend powders, respectively.

#### Blood sample collection and analyses

Ten milliliters of arterialized venous blood was collected into a syringe at each time point. Five milliliters of that sample was added to EDTA-containing tubes (BD vacutainer LH; BD Diagnostics, Nu-Care) and centrifuged for 10 min at 4000 × *g* at 4°C. The plasma supernatant was then removed, aliquoted, and stored at −80°C for later analyses. The remaining 5 mL of blood was added to additional vacutainers (BD vacutainers SST II, BD Diagnostics, Nu-Care) and left upright to clot at room temperature for 30 min and then centrifuged for 10 min at 4000 × *g* at 4°C. The serum supernatant was then removed, aliquoted, and stored at −80°C for future analyses.

Serum insulin concentrations were measured using a commercially available ELISA kit (DRG Insulin ELISA, EIA-2935, DRG International Inc). Concentrations of glycine, phenylalanine, leucine, valine, isoleucine, lysine, histidine, glutamic acid, methionine, proline, serine, threonine, tyrosine, and alanine were determined as tert-butyldimethylsilyl derivatives by GC-MS with electron impact ionization (Agilent), as described previously [43]. Briefly, to prepare samples for GC-MS, 10 μL of 2 mM nor-leucine was added as an internal standard to 450 μL plasma and deproteinized on ice with 450 μL of 15% w/v 5-sulfosalcylic acid. Samples were then vortexed and centrifuged at 4000 × *g* for 10 min at 4°C. The supernatant was then loaded onto cation exchange columns. Columns were washed with ddH_2_O, followed by 6 mL 0.5 M acetic acid and a further wash with ddH_2_O, with the columns allowed to drain between each step. The amino acids were then eluted with 2 mL of 6 M ammonia hydroxide (NH_4_OH). The eluate was dried using a Speed-Vac (Thermo Fisher Scientific) for 8 h at 60°C before being derivatized and analyzed by GC-MS, as described previously [43].

#### Statistical analyses

A 2-sided power analysis with expected effect sizes estimated from previous research [15,18] revealed that *n* = 8 was sufficient to detect differences in postprandial amino acid availability (*P* < 0.05, power 80%; G∗Power version 3.1.9.2). Factoring a 20% drop out rate, 10 participants were recruited with 1 dropout, leaving a total *n* = 9. Statistical significance was set at *P* < 0.05. All calculations were performed with GraphPad 7.1. All data are expressed as mean and SEM. Postprandial plasma amino acid, glucose, and insulin availability (incremental AUC [iAUC]) were compared using paired samples *t* test. Postprandial plasma amino acid, glucose, and insulin kinetics were analyzed using 2-way analysis of variance (ANOVA; condition × time). Resting energy expenditure data were analyzed using 2-way ANOVA (condition × time). VAS appetite scores were expressed as change from baseline and analyzed using 2-way ANOVA (condition × time).

### Study B

#### INFOGEST static in vitro digestion method

In vitro digestion was determined using the INFOGEST model as previously described [35]. Both CON and EXT were put through the static model in duplicate. Simulated salivary fluid (SSF), simulated gastric fluid (SGF), and simulated intestinal fluid (SIF) were prepared, as previously described [44]. Protein powder (0.3 g) was added to 4.4 mL ddH_2_O in a 50-mL centrifuge tube in order to simulate the beverage used in Study A. First, oral phase digestion involved the addition of 4.23 mL SSF, 23.5 μL CaCl_2_, and 0.446 mL ddH_2_O. α-salivary amylase was not included in this step as starch digestion was not an outcome of the present work.

Second, to simulate the gastric phase, 7.52 mL SGF, 4.7 μL CaCl_2_, 0.625 mL ddH_2_O, 0.31 mL 1 M HCl, and pepsin (2000 U/mL) (Merck, P6887) were added to the CON and EXT samples. Samples were incubated (37°C, pH 3) and rotated (70 × *g*) for 120 min. Aliquots (200 μL) were collected at baseline (before addition of pepsin) and 1, 2, 5, 10, 15, 30, 60, 90, and 120 min following the addition of pepsin. The enzymatic reaction for the gastric phase was stopped by the addition of 1 M NaOH to elevate the pH >7.2.

Third, for the intestinal phase, 10.36 mL SIF, 29.6 μL CaCl_2_, 2.76 mL ddH_2_O, 140 μL 1 M NaOH, trypsin (100 U/mL) (Merck, T0303), and chymotrypsin (25 U/mL) (Merck, C4129) were added to the CON digesta. To the EXT digesta was added 10.36 mL SIF, 29.6 μL CaCl_2_, 2.78 mL ddH_2_O, 100 μL 1 M NaOH, trypsin, and chymotrypsin. Samples were incubated (37°C, pH 7) and rotated (70 × *g*) for 120 min. Aliquots (200 μL) were collected from each digesta at baseline and 1, 2, 5, 10, 15, 30, 60, 90, and 120 min following the addition of trypsin and chymotrypsin. The enzymatic reaction for the intestinal phase was stopped by the addition of a serine proteinase inhibitor, pefabloc (Merck, 76307).

An additional sample of digesta (1 mL) was collected at baseline and at the end of each phase for further microscopy analysis. Following collection, samples were immediately stored at −20°C for subsequent analysis.

#### Protein digesta analysis

Protein concentration of the digesta during digestion was quantified by bicinchoninic acid (BCA) assay (Abcam, ab287853) as previously described [45]. Briefly, 10 μL of standard or sample were incubated with 200 μL working reagent for 30 min at 37°C. Absorbance was then measured spectrophotometrically at 562 nm (Benchmark Plus spectrophotometer; Bio-Rad Laboratories, Inc.). All samples were performed in duplicate in a 96-well plate across 2 separate measures.

SDS-PAGE was performed to assess the digestion pattern of the different proteins (MYC/PP) and their subfractions at each time point. First, samples were diluted (75 μL sample + 925 μL ddH_2_O). The diluted sample was then added to 10 μL NuPAGE reducing agent (Bio-Rad Laboratories, Inc.) and 25 μL NuPAGE lithium dodecyl sulfate sample buffer (Bio-Rad Laboratories, Inc.) before being vortexed and heated at 70°C for 10 min. Once cooled, 15 μL of sample were loaded onto the precast gel (Bio-Rad Laboratories, Inc.). NuPAGE MES buffer (Bio-Rad Laboratories, Inc; 50 mL MES 20× + 950 mL ddH_2_O) was then added to the electrophoresis unit, and the gel was run for 35 min at 200 V, 350 mA, 100 W (Bio-Rad Laboratories, Inc.). Thereafter, the gel was removed and incubated overnight in fixing solution (50% methanol, 10% acetic acid). The gel was then rinsed with ddH_2_O to remove any residual fixing solution before being added to 50 mL Coomassie blue stain (Thermo Fisher, LC6060) for incubation overnight. The gel was then rinsed again with ddH_2_O before being scanned (GS-800 calibrated densitometer) and imaged (Quantity One 1-D analysis software version 1).

#### Fluorescence microscopy imaging

Fluorescence microscopy samples were not separated (to soluble and insoluble fractions) prior to imaging, so images were captured of the total digesta. Thirty microliters of sample were added to 300 μL of phosphate-buffered saline (PBS) in a 1-mL microcentrifuge tube. Next, neat calcofluor white (CW; Sigma 18909; 1:100) and 1 mg/mL fast green (FG; Sigma F7252; 1:50) solutions were added to the PBS mixture. Following 30 min incubation, the tubes were centrifuged (3.5 × *g*) for 2 min before adding 15 μL of the stained sample to the microscope slide and mounting with a 1.5 cm coverslip. Samples were imaged by confocal microscopy using the Zeiss LSM 880 Airyscan Confocal Microscope in line scanning mode using ZEN Black software with ×10/0.5 numeric aperture [NA], ×20/0.8 NA, and ×40/1.1 NA objectives. Excitation and emission for CW were 405 nm and 410–524 nm, respectively. Excitation and emission for FG were 561 nm and 568–712 nm, respectively. Negative controls, which were examined without the presence of the CW and FG stains, were set up in parallel. From these controls, we were able to determine that autofluorescence in the samples was extremely low.

## Results

### Study A

#### Serum insulin concentrations

**FIGURE 2:**
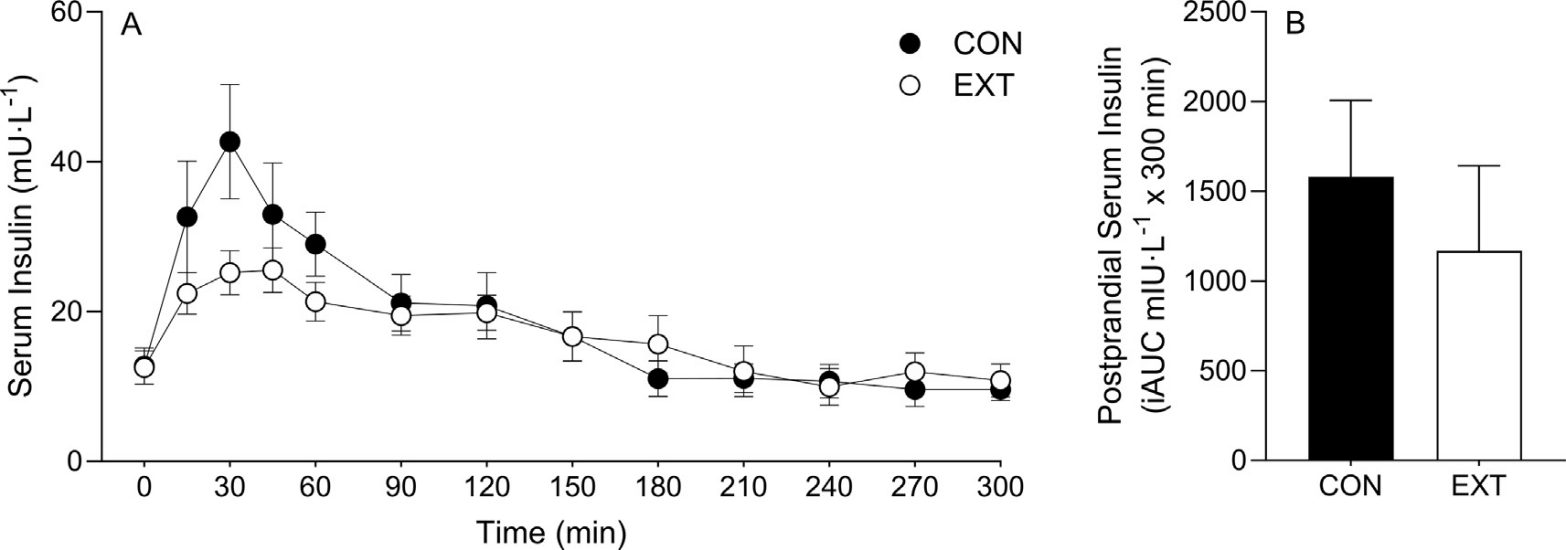
Time course (A) and incremental area under the curve (B) (incremental area under the curve [iAUC]; calculated as above *t* = 0) of serum insulin concentrations for a 5-h postprandial period. Experimental beverage consisting of 39%/61% mycoprotein/pea protein (31.9 g of dry blend containing 25 g protein [CON] or 31.4 g of extruded blend containing 25 g protein [EXT]) consumed at *t* = 0. Time course data were analyzed using a 2-way repeated measures analysis of variance (condition × time) with Sidak post hoc tests to detect differences at individual time points. iAUC data were analyzed using a paired *t* test. Time effect; *P* < 0.05. Condition effect; *P* > 0.05. Condition × time interaction; *P* < 0.001. Values are mean ± standard error of the mean.

Serum insulin concentrations over the time course of the experiment are depicted in Figure 2 (*n* = 9). Fasting serum insulin concentrations were similar between conditions (CON, 13 ± 2 mU/L; EXT, 13 ± 2 mU/L; *P* > 0.05). The ingestion of the protein blends increased serum insulin concentrations (time effect, *P* < 0.0001) and to a different extent between conditions (time × condition interaction; *P* < 0.001). Despite these divergent temporal responses, serum insulin concentrations did not differ between groups at any specific time point, and there was no difference in postprandial serum insulin iAUC between conditions (*P* > 0.05).

#### Plasma amino acid concentrations

**FIGURE 3:**
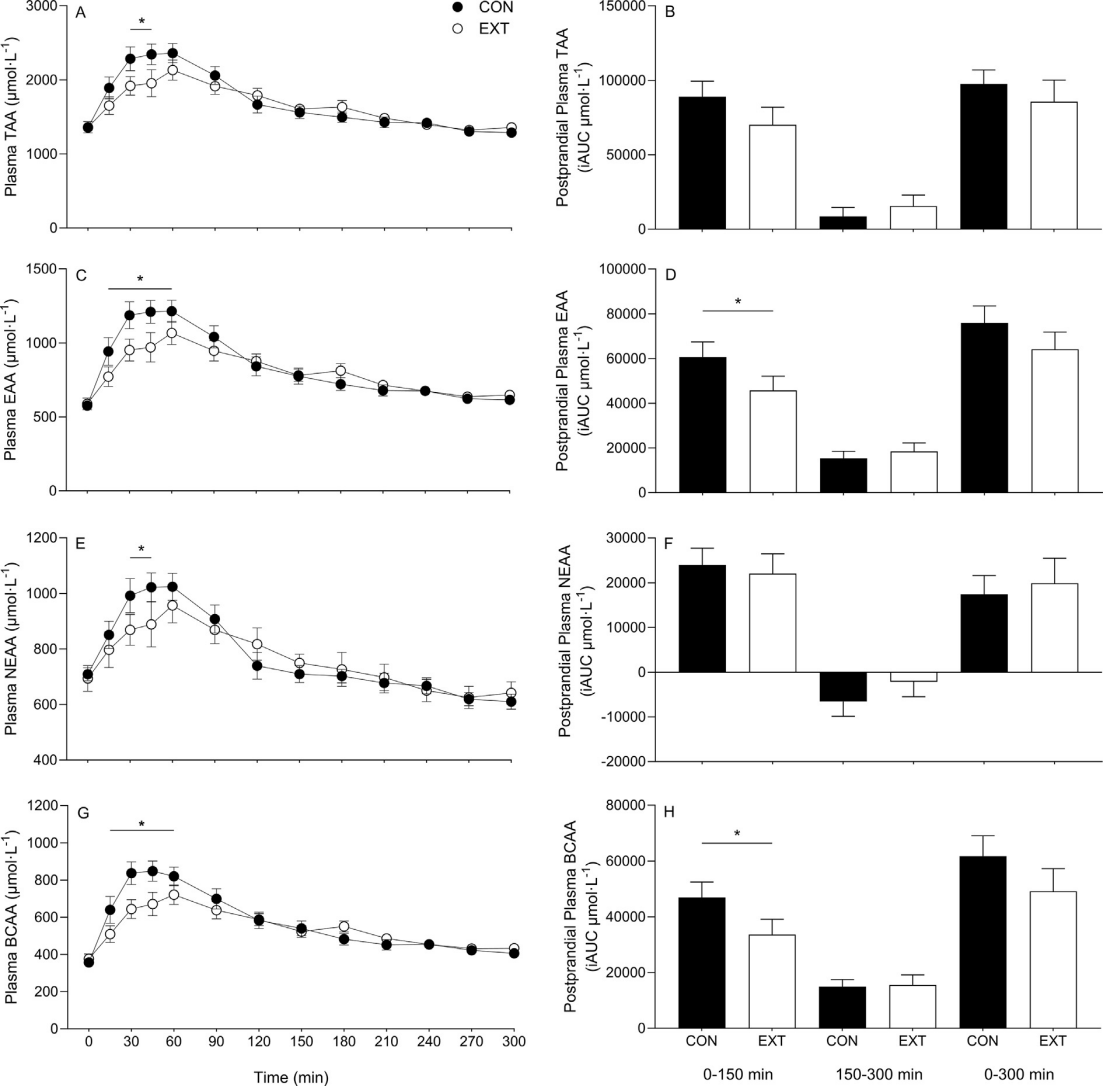
Time course and incremental area under the curve (incremental area under the curve [iAUC]; calculated as above *t* = 0) of plasma total amino acids (TAAs) (A, B), essential amino acids (EAAs) (C, D), nonessential amino acids (NEAAs) (E, F), and branched-chain amino acids (BCAAs) (G, H) over a 5-h postprandial period. iAUCs are displayed for early (0–150 min), late (150–300 min), and total (0–300 min) postprandial periods. Experimental beverage consisting of 39%/61% mycoprotein/pea protein (31.9 g of dry blend containing 25 g protein [CON] or 31.4 g of extruded blend containing 25 g protein [EXT]) consumed at *t* = 0. Time course data were analyzed using a 2-way repeated measures analysis of variance (condition × time) with Sidak post hoc tests to detect differences at individual time points. iAUC data were analyzed using a paired *t* test. ∗ denotes individual differences between groups at that time point and a difference between conditions on the bar graphs (*P* < 0.05). Time effect; all *P* < 0.05. Condition effect; all *P* > 0.05. Condition × time interaction; all *P* < 0.001). Values are mean ± standard error of the mean.

**FIGURE 4:**
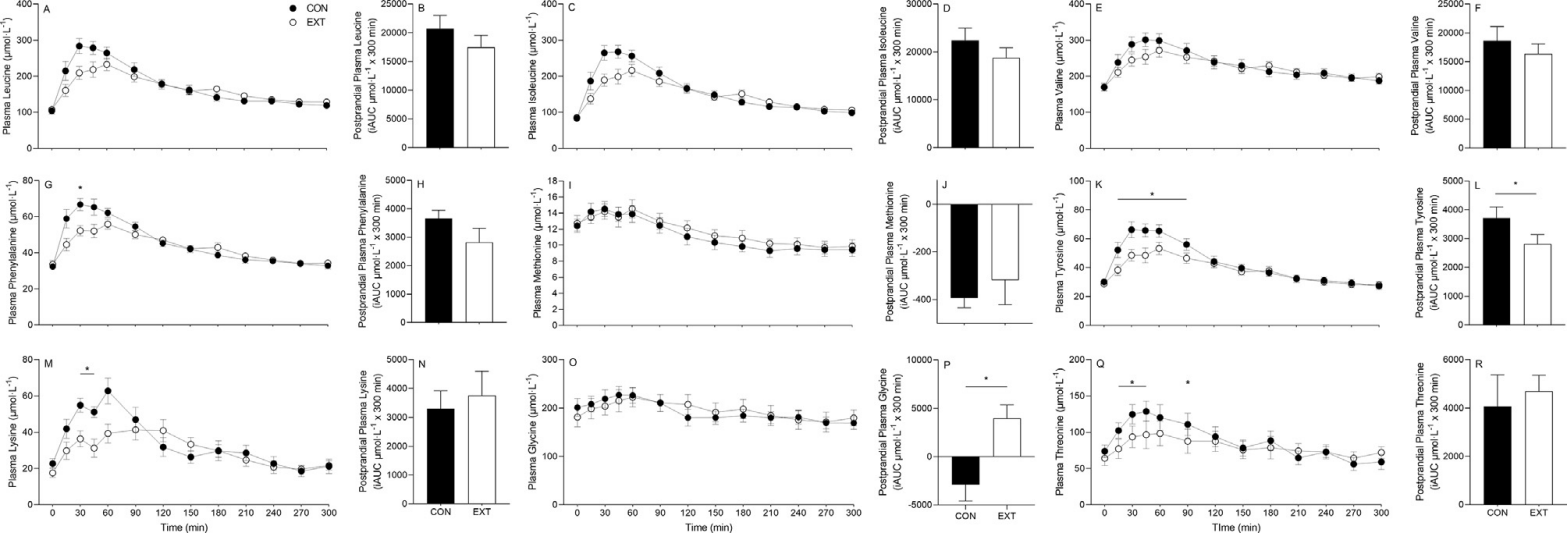
Time course and incremental area under the curve (iAUC; calculated as above *t* = 0) of plasma leucine (A, B), isoleucine (C, D), valine (E, F), phenylalanine (G, H), methionine (I, J), tyrosine (K, L), lysine (M, N), glycine (O, P), and threonine (Q, R) over a 5-h postprandial period. Experimental beverage consisting of 39%/61% mycoprotein/pea protein (31.9 g of dry blend containing 25 g protein [CON] or 31.4 g of extruded blend containing 25 g protein [EXT]) consumed at *t* = 0. Time course data were analyzed using a 2-way repeated measures analysis of variance (condition × time) with Sidak post hoc tests to detect differences at individual time points. iAUC data were analysed using a paired *t* test. ∗ denotes individual differences between groups at that time point and a difference between conditions on the bar graphs (*P* < 0.05). Time effect; all *P* < 0.05. Condition effect; all *P* > 0.05 with the exception of tyrosine, phenylalanine, and lysine (*P* < 0.05). Condition × time interaction; all *P* < 0.05 with the exception of glycine and methionine (*P* > 0.05). Values are mean ± standard error of the mean.

Plasma total (TAA), essential (EAA), nonessential (NEAA), branched-chain (BCAA), and individual amino acid concentrations over the time course of the experiment are presented in FIGURE 3, FIGURE 4. All plasma amino acid concentrations increased (with the exception of methionine, which decreased) following protein blend ingestion (time effects, all *P* < 0.01). Plasma TAAs, EAAs, NEAAs, and BCAAs all displayed a more rapid increase following ingestion of CON compared with EXT (condition × time interactions, all *P* < 0.001). Plasma TAA and NEAA concentrations were greater at 30 and 45 min in the CON compared with EXT condition (all *P* < 0.05). Plasma EAA and BCAA concentrations were elevated in the CON compared with EXT condition at 15, 30, 45, and 60 min (all *P* < 0.05). All individual amino acids displayed a more rapid increase following ingestion of CON compared with EXT (condition × time interaction, *P* < 0.05) with the exceptions of glycine, histidine, glutamic acid, methionine, proline, and alanine, for which there were no differences between conditions (*P* > 0.05) (data not shown for proline, alanine, glutamine, and histidine). Plasma concentrations of phenylalanine were greater in the CON condition at 30 min (*P* < 0.05). Plasma lysine concentrations were elevated compared with EXT at 30 and 45 min (*P* < 0.05). Greater concentrations of plasma threonine and tyrosine concentrations were observed in the CON condition at their respective time points (*P* < 0.05). Table 3 displays peak concentrations and time to peak (TTP) concentrations for all amino acids measured and grouped amino acids. Lower peak concentrations were observed in the EXT condition for glycine, leucine, isoleucine, phenylalanine, lysine, threonine, tyrosine, EAAs, and BCAAs (*P* < 0.05), with trends for lower peaks in histidine, serine, and TAAs (*P* < 0.1). Delayed TTP was observed for phenylalanine, serine, and NEAAs (*P* < 0.05), with trends for delayed TTP in glycine, leucine, isoleucine, lysine, threonine, tyrosine, TAAs, EAAs, and BCAA (*P* < 0.1).

**TABLE 3:** Peak concentration and time to peak concentration for all amino acids measured

	CON	EXT	sig.
Glycine			
Peak (μmol/L)	244 ± 16	239 ± 19	0.034
Time to peak (min)	68 ± 18	95 ± 28	0.062
Valine			
Peak (μmol/L)	314 ± 17	281 ± 16	0.119
Time to peak (min)	50 ± 3	80 ± 18	0.172
Leucine			
Peak (μmol/L)	297 ± 18	240 ± 16	0.019
Time to peak (min)	38 ± 4	73 ± 15	0.050
Isoleucine			
Peak (μmol/L)	283 ± 16	223 ± 15	0.019
Time to peak (min)	40 ± 3	73 ± 15	0.059
Phenylalanine			
Peak (μmol/L)	72 ± 3	58 ± 3	0.020
Time to peak (min)	32 ± 4	83 ± 20	0.037
Lysine			
Peak (μmol/L)	66 ± 6	47 ± 5	0.002
Time to peak (min)	43 ± 5	82 ± 18	0.096
Histidine			
Peak (μmol/L)	139 ± 11	124 ± 10	0.068
Time to peak (min)	68 ± 17	75 ± 17	0.825
Glutamic acid			
Peak (μmol/L)	187 ± 15	158 ± 9	0.152
Time to peak (min)	45 ± 3	45 ± 5	0.999
Methionine			
Peak (μmol/L)	15 ± 1	15 ± 1	0.985
Time to peak (min)	33 ± 6	43 ± 6	0.219
Proline			
Peak (μmol/L)	60 ± 10	59 ± 11	0.583
Time to peak (min)	60 ± 8	112 ± 24	0.133
Serine			
Peak (μmol/L)	223 ± 19	190 ± 17	0.061
Time to peak (min)	41 ± 6	95 ± 21	0.035
Threonine			
Peak (μmol/L)	146 ± 11	114 ± 15	0.004
Time to peak (min)	67 ± 14	112 ± 25	0.069
Tyrosine			
Peak (μmol/L)	71 ± 4	55 ± 4	0.019
Time to peak (min)	42 ± 4	75 ± 14	0.062
Alanine			
Peak (μmol/L)	339 ± 22	326 ± 33	0.661
Time to peak (min)	50 ± 3	63 ± 8	0.154
TAA			
Peak (μmol/L)	2525 ± 107	2209 ± 126	0.067
Time to peak (min)	45 ± 5	88 ± 19	0.063
EAA			
Peak (μmol/L)	1298 ± 68	1100 ± 72	0.042
Time to peak (min)	43 ± 5	87 ± 20	0.076
NEAA			
Peak (μmol/L)	1096 ± 36	995 ± 59	0.156
Time to peak (min)	47 ± 6	98 ± 20	0.046
BCAA			
Peak (μmol·L^-1^)	890 ± 49	743 ± 47	0.035
Time to peak (min)	42 ± 4	75 ± 14	0.062

Abbreviations: BCAA, branched-chain amino acid; CON, dry blend; EAA, essential amino acid; EXT, extruded blend; NEAA, nonessential amino acids; SEM, standard error of the mean; sig., significance; TAA, total amino acid.

Peak and time to peak were compared using paired samples *t* tests. Values are mean ± SEM.

Total postprandial amino acid availabilities for TAAs, EAAs, NEAAs, and BCAAs and individual amino acids, as indicated by iAUC, are also displayed in FIGURE 3, FIGURE 4 (inset graphs). Temporal postprandial amino acid iAUCs (early, late, total) are also displayed for TAAs, EAAs, NEAAs, and BCAAs (Figure 3). Plasma amino acid availability over the total postprandial period was 28% greater in the CON compared with EXT condition for tyrosine (*P* < 0.05), with trends (*P* < 0.1) for greater availability of leucine (17%), isoleucine (18%), and BCAAs (23%), whereas plasma glycine availability was 199% lower in CON (*P* < 0.001), and no differences were observed for the remaining amino acids (all *P* > 0.05). Postprandial plasma availability of EAAs and BCAAs in the early (0–150 min) period was 28% and 33% greater in the CON compared with EXT condition, respectively. There was also a trend (*P* = 0.06) for greater plasma TAA availability over the early postprandial period in the CON compared with the EXT condition. No differences in availability were observed for any amino acid parameter in the latter postprandial period (150–300 min).

#### Whole-body energy expenditure and appetite

TEE (expressed as kilojoules per day) in the postabsorptive and postprandial (60, 120, 180, 240, 300 min) states is displayed in Table 4. Postabsorptive TEE did not differ between conditions (*P* > 0.05). The ingestion of protein resulted in an increase in TEE (time effect, *P* < 0.001) and by the same extent in both conditions (condition × time interaction, *P* > 0.05).

**TABLE 4:** Whole-body resting energy expenditure and VAS appetite scores over the course of the experimental trial following the ingestion of the CON and EXT blend

Min	Energy expenditure (kJ/d)		Appetite score (VAS)	
	CON	EXT	CON	EXT
Basal	6449 ± 546	6656 ± 520	59 ± 4	52 ± 6
5			36 ± 7	35 ± 7
60	7271 ± 531	7266 ± 583	45 ± 4	46 ± 6
120	6774 ± 515	7050 ± 645	52 ± 6	56 ± 5
180	6600 ± 527	7138 ± 538	63 ± 5	65 ± 4
240	6523 ± 555	7086 ± 487	71 ± 5	68 ± 4
300	6254 ± 413	6898 ± 348	75 ± 5	74 ± 4

Abbreviations: CON, dry blend; EXT, extruded blend; VAS, visual analog scale.

Values are mean ± SEM. Lower VAS score is associated with a lower appetite. Significant time effect (*P* < 0.001) for energy expenditure and appetite score; no significant differences between conditions at any time point (*P* > 0.05).

Appetite scores as measured using VASs are displayed in Table 4. Appetite scores did not differ between conditions in the postabsorptive state (*P* > 0.05), but protein blend ingestion decreased and reincreased appetite (time effect, *P* < 0.0001) to the same extent in both conditions (condition × time interaction, *P* > 0.05).

### Study B

**FIGURE 5:**
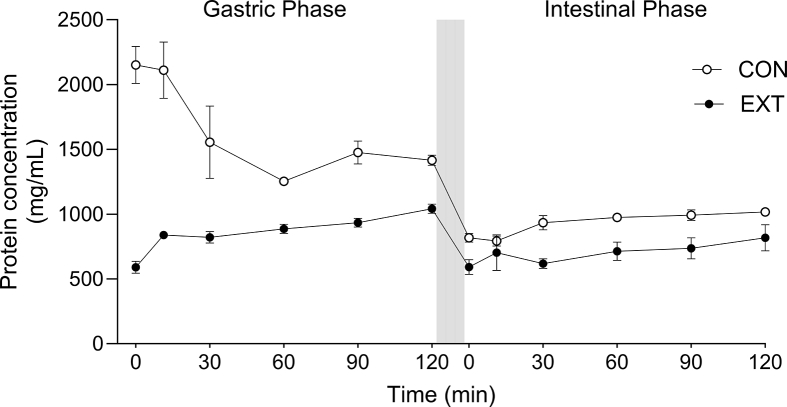
Time course of protein concentrations in the digesta measured via BCA assay for CON and EXT (both 0.3 g protein powder assimilated in 4.4 mL of ddH_2_O) during the INFOGEST static in vitro digestion (2 h gastric and 2 h intestinal phase). Data were analyzed using a 2-way ANOVA (condition × time) with Sidak post hoc tests to detect differences at individual time points. Time × condition interaction (*P* < 0.0001) during the gastric phase. Condition effect (*P* < 0.001) during the intestinal phase. Values are mean (*n* = 6) ± SEM. BCA, bicinchoninic acid; CON, dry blend; EXT, extruded blend; SEM, standard error of the mean.

Figure 5 reports the protein content in the digesta throughout the gastric and intestinal phases of digestion. Protein solubility in the undigested sample was higher in the CON compared with EXT (CON, 2150 ± 129 mg/mL; EXT, 590 ± 41 mg/mL; ∼113% difference) (condition effect, *P* < 0.0001). This is unlikely to reflect lower *actual* protein content in the EXT sample, as independent protein and amino acid content for the protein sources were well matched (Table 2). Rather, this difference is likely attributed to HME-induced aggregation in the EXT sample, which impaired the ability of protein quantification assays (including the BCA assay) to accurately detect protein concentrations [46].

Following addition of pepsin, the protein content decreased in the CON condition only (time effect, *P* < 0.01) but remained ∼64% higher than the EXT condition throughout the gastric phase (time × condition interaction, *P* < 0.0001). Protein concentrations remained ∼30% higher in the CON compared with EXT condition throughout the intestinal phase (condition effect, *P* < 0.001), whereas the addition of trypsin and chymotrypsin did not affect protein concentrations in either condition (time × condition interaction, *P* > 0.05).

**FIGURE 6:**
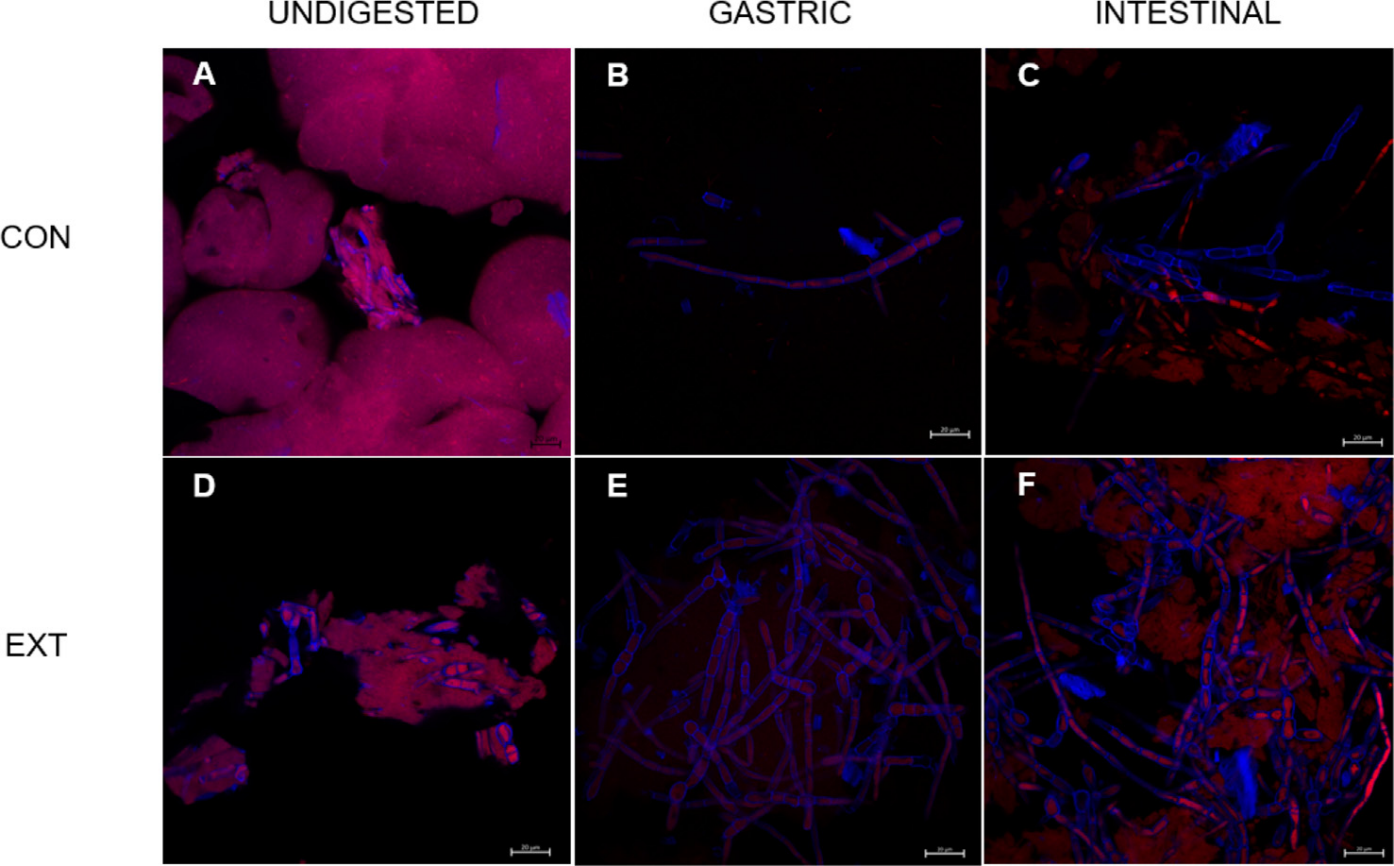
Optical micrographs obtained using fluorescence microscopy with fast green (FG; stained as pink/red) and calcofluor white (CW; stained as a brilliant blue) stains for protein and cell wall, respectively, in CON and EXT conditions. Images are presented from before (A, D) and after gastric (B, E) and intestinal (C, F) INFOGEST static in vitro digestion. Protein is visibly more abundant in the CON compared with EXT condition in the undigested sample. The darker red and more clustered protein structures in the undigested EXT sample indicate dense aggregate structures. This likely underpins the rapid and greater protein availability observed with CON measured both in vivo and in vitro. The free protein that existed in the CON sample has been digested by the end of the gastric phase, leaving only the protein that is found within the hyphal structure of mycoprotein, which is liberated from the cell wall and digested during the intestinal phase. Conversely, dense protein aggregates can still be observed in the EXT sample following intestinal digestion, supporting the impaired digestibility of the EXT. CON, dry blend; EXT, extruded blend.

To explain the quantitative in vivo and in vitro data, we used SDS-PAGE (Supplementary Figure 2) and fluorescence microscopy (Figure 6) to provide a subjective explanation and further interpretation of our data. The quantitative protein concentration data were corroborated by the SDS-PAGE data, which showed visibly more protein in the CON compared with the EXT condition from the outset. This resulted in clear differences in the amount of protein visible during the gastric phase of digestion between the 2 conditions. Optimal micrographs obtained by fluorescence microscopy show the protein matrix structure in the undigested state and following gastric and intestinal digestion (Figure 6). In the undigested state, images show an abundance of free protein surrounding the hyphal MYC structures in the CON sample. The majority of the free protein had been digested as it was no longer visible by the end of the gastric phase. At the end of the intestinal phase, the remaining protein that was previously within the hyphal structure had been released. In comparison, the EXT sample had much less free protein and had instead formed dense protein aggregates, and the hyphal structure of MYC was fragmented. By the end of the gastric phase, what little free protein existed had been digested, leaving the protein within the MYC. Similar to the CON, the protein within the hyphal structure had been released by the end of the intestinal phase with a high number of proteinless hyphae cell wall structures now present, although there was clearly still more visible (undigested) protein present at the end of each digestion phase in the EXT compared with the CON condition.

## Discussion

The present study used a multidisciplinary approach to comprehensively assess whether HME influences in vivo systemic amino acid availability and/or in vitro bioaccessibility of a dietary protein blend following its ingestion. In support of our hypothesis, we observed reduced postprandial plasma amino acid concentrations following ingestion of an extruded (EXT) compared with nonextruded (CON) protein blend in healthy humans. These in vivo observations are supported by our in vitro findings revealing lower protein digestibility of the EXT during the gastric phase, which is likely attributable to HME-induced aggregate formation within the PP fraction of the blend.

The form in which we ingest dietary protein is thought to affect postprandial amino acid availability and, therefore, the metabolic fate of those amino acids [[10], [11], [12], [13], [14]]. HME is a commonly used industrial food process typically used to produce meat alternative products, as it promotes chemical and physical alterations in the protein structure that allow blending and texturization of different alternative protein sources [20]. To date, research has primarily focused on how these changes affect texture and sensory outcomes [47], with a small amount of research with in vitro models investigating how HME affects solubility and digestibility [[25], [26], [27], [28], [29], [30]], but human data concerning the impact on postprandial amino acid bioavailability are lacking. In Study A of the present work, we report that HME resulted in delayed plasma amino acid availability following ingestion of a MYC/PP blend (FIGURE 3, FIGURE 4). Specifically, we observed earlier and greater peaks, and more sustained elevations, of plasma TAA, EAA, NEAA, and BCAA concentrations following ingestion of the CON compared with an identical protein that had undergone HME (EXT). This resulted in lower total plasma availability (as indicated by iAUC) of EAAs and BCAAs over the first 150 min of the postprandial period in the EXT condition, an effect that was compensated for over the later (150–300 min) postprandial period. Our data are in line with other studies that report various food processing techniques can alter postprandial plasma amino acid kinetics [15,16,18,48]. For example, disrupting the micellar casein structure through acidification or enzymatic reaction can impair and increase peak amino acid concentrations in plasma, respectively [18]. Furthermore, the glycation of milk protein has been demonstrated to attenuate the postprandial increase in plasma EAA concentrations [15]. Clearly, the various processing methods frequently used to produce the dietary protein we eat in day-to-day life can have profound effects on the protein structure. These structural changes can influence the postprandial amino acid availability and, likely, the consequent metabolic fate of the ingested amino acids. For example, faster and greater rises in postprandial plasma amino acid concentrations have been demonstrated to result in a greater increase in amino acid oxidation [49] and are often suggested to produce a greater anabolic response [5]. Furthermore, the majority of dietary protein we ingest is contained within nutrient-dense, structurally complex food/protein matrices that have undergone (multiple) processing methods. Therefore, expanding our understanding of how nutrient-dense whole foods, food processing, and the interaction between these factors regulate postprandial protein metabolism is clearly a research priority.

The degree of postprandial plasma aminoacidemia can be modulated by postdigestive processes including splanchnic extraction, peripheral amino acid uptake, and muscle protein breakdown [50]. However, we reasoned HME would likely bring about modified postprandial plasma amino acid responses as a result of alterations in the structural complexity of the protein blend, thereby affecting motility at different stages of digestion. To overcome technical challenges associated with studying discrete aspects of digestion and absorption in vivo*,* we employed an established and standardized in vitro approach [35] to complement and explain our in vivo data. In Study B we observed striking differences in (particularly gastric phase) digestion between the CON and EXT protein blends. Figure 5 illustrates the 113% higher protein availability (i.e., bioaccessibility) in the CON compared with the EXT condition in the undigested sample, despite these samples being isonitrogenous and closely matched for amino acid content. Simulated gastric digestion rapidly reduced protein concentrations in the CON condition, indicative of pepsin-mediated protein degradation to dipeptides/free amino acids (simpler molecules below the detection limits of a BCA assay). Therefore, the observed decrease in protein concentrations in the CON condition reflects the natural digestive process of releasing amino acids, allowing more rapid intestinal absorption. This effect also appeared evident within Study A, underpinning the faster postprandial rise in systemic amino acids. Conversely, not only did HME reduce protein availability prior to digestion, this remained the case throughout gastric and intestinal phases of digestion. Therefore, these effects clearly offer a mechanistic explanation for the delayed postprandial systemic amino acid availability observed with EXT in Study A.

In an attempt to understand the role of HME in altering the structural complexity of the protein blends, microscopy images were taken from samples in the undigested state and revealed remarkable differences between the 2 blends (Figure 6). First, the hyphal structure of MYC [37] can be seen in the undigested CON and EXT samples, albeit slightly fragmented in the EXT sample. Despite this damage, however, it is unlikely to have disrupted the cell wall as it is remarkably resistant to physical and chemical stress [51]. Furthermore, the protein within MYC is predominantly digested in the small intestine, as intestinal proteases diffuse across the (intact) fungal cell wall to hydrolyze the intracellular protein [51]. Accordingly, it is unlikely that the differences in gastric phase digestion are attributable to impairments in MYC digestibility. Rather, free protein observed in the undigested CON sample is likely primarily derived from PP. In support of the in vitro protein availability data (Figure 5), the EXT sample shows far more dense protein aggregates, which reduce protein availability and accessibility (surface area) to proteolytic enzymes. This implies that the impairment in gastric digestion and plasma amino acid bioavailability we report across both the present experimental approaches are explained by HME-induced aggregate formation within PP. In agreement with this, HME of PP has previously been demonstrated to cause aggregate formation, rendering it resistant to pepsin hydrolysis [30]. We therefore corroborate previous research consistently reporting impairments in digestibility of PP following HME using in vitro models [29,30]. However, we also extend on this by providing a plausible explanation for how the structural complexity of a protein source can be altered by HME, resulting in impaired gastric phase digestion and delayed postprandial amino acid bioavailability.

Collectively, data discussed above from Studies A and B point to the conclusion that HME reduces the nutritional value of dietary protein. This might have practical implications for nutritional advice, especially protein recommendations to populations for whom more processed foods are the norm or where protein intake is already of concern (e.g., older adults). However, it is important to note that (aspects of) such findings are not uniformly observed across different protein sources in similar studies, as some experiments have reported improved protein digestibility following HME [[25], [26], [27]]. So far, the structural changes that occur during HME are difficult to understand [24] or quantify; however, advances in computer simulation technology can improve our ability to characterize, and eventually predict, the behavior of protein during the HME process [20]. Nevertheless, it is well known that plant proteins, including pea globulins, will denature and unfold during heating, exposing previously hidden hydrophobic residues, leading to aggregation [52] and gelation [53]. Once the HME process and its effects on a variety of food structures are better understood and appreciated, it will offer the ability to manipulate food structures and their behavior through the GI tract. Combining these technological advances with investigations into how such structural changes affect human metabolism offers an exciting tool in the drive for a sustainable, flexible, and healthy future foods landscape.

In conclusion, HME delays in vivo postprandial systemic amino acid bioavailability in human volunteers and impairs in vitro digestibility of a non–animal-derived (MYC/PP) protein blend. This impairment in digestion appears to be primarily caused by aggregate formation in PP, reducing the bioaccessibility of the protein to proteolytic enzymes throughout the GI tract. These data illustrate how industrial processing techniques to drive product development and innovation can introduce a level of structural complexity to a protein source that may influence postprandial metabolism of dietary protein in vivo.

## Author contributions

The authors’ responsibilities were as follows – SW, BTW, AJM, FBS, PJW: designed the research; SW, BTW, FBS, GM: conducted the research; SW, DRA, CB: performed the analysis; GW: was responsible for randomization and preparation of the experimental beverages; SW, BTW: analyzed the data and wrote the manuscript; BTW: had primary responsibility for the final content; and all authors: read and approved the final manuscript.

### Conflict of interest

TJA Finnigan was an employee of Marlow Foods; BTW, AJM, and FBS are employees of the University of Exeter. All other authors report no conflicts of interest.

## Funding

The project was funded by Marlow Foods Ltd (BTW as grant holder). The private partners have contributed to the project through regular discussion. SW and GW were supported by a studentship in collaboration with Marlow Foods Ltd. AJM and DRA are supported in part by a grant from the National Institute of Aging (P30-AG024832). SW is now supported by the Oxford National Institute for Health and Care Research (NIHR) Biomedical Research Centre. The views are those expressed by the author(s) and not necessarily those of the NIHR.

## Data availability

Data described in the manuscript may be made available upon request, pending application.

## Appendix A Supplementary data

Multimedia component1 Multimedia component1

## References

[1] van der Heijden I., Monteyne A.J., Stephens F.B., Wall B.T. (2023). Alternative dietary protein sources to support healthy and active skeletal muscle aging. Nutr. Rev..

[2] Burd N.A., McKenna C.F., Salvador A.F., Paulussen K.J.M., Moore D.R. (2019). Dietary protein quantity, quality, and exercise are key to healthy living: a muscle-centric perspective across the lifespan. Front. Nutr..

[3] Morgan P.T., Harris D.O., Marshall R.N., Quinlan J.I., Edwards S.J., Allen S.L. (2021). Protein source and quality for skeletal muscle anabolism in young and older adults: a systematic review and meta-analysis. J. Nutr..

[4] Burd N.A., Yang Y., Moore D.R., Tang J.E., Tarnopolsky M.A., Phillips S.M. (2012). Greater stimulation of myofibrillar protein synthesis with ingestion of whey protein isolate v. micellar casein at rest and after resistance exercise in elderly men. Br. J. Nutr..

[5] Pennings B., Boirie Y., Senden J.M.G., Gijsen A.P., Kuipers H., van Loon L.J. (2011). Whey protein stimulates postprandial muscle protein accretion more effectively than do casein and casein hydrolysate in older men. Am. J. Clin. Nutr..

[6] Gorissen S.H.M., Crombag J.J.R., Senden J.M.G., Waterval W.A.H., Bierau J., Verdijk L.B. (2018). Protein content and amino acid composition of commercially available plant-based protein isolates. Amino Acids.

[7] Gorissen S.H.M., Trommelen J., Kouw I.W.K., Holwerda A.M., Pennings B., Groen B.B.L. (2020). Protein type, protein dose, and age modulate dietary protein digestion and phenylalanine absorption kinetics and plasma phenylalanine availability in humans. J. Nutr..

[8] Tang J.E., Moore D.R., Kujbida G.W., Tarnopolsky M.A., Phillips S.M. (2009). Ingestion of whey hydrolysate, casein, or soy protein isolate: effects on mixed muscle protein synthesis at rest and following resistance exercise in young men. J. Appl. Physiol..

[9] van Vliet S., Shy E.L., Abou Sawan S., Beals J.W., West D.W., Skinner S.K. (2017). Consumption of whole eggs promotes greater stimulation of postexercise muscle protein synthesis than consumption of isonitrogenous amounts of egg whites in young men. Am. J. Clin. Nutr..

[10] West S., Monteyne A.J., Whelehan G., Abdelrahman D.R., Murton A.J., Finnigan T.J.A. (2023). Mycoprotein ingestion within or without its wholefood matrix results in equivalent stimulation of myofibrillar protein synthesis rates in resting and exercised muscle of young men. Br. J. Nutr..

[11] Pennings B., Groen B.B.L., van Dijk J.W., de Lange A., Kiskini A., Kuklinski M. (2013). Minced beef is more rapidly digested and absorbed than beef steak, resulting in greater postprandial protein retention in older men. Am. J. Clin. Nutr..

[12] Evenepoel P., Geypens B., Luypaerts A., Hiele M., Ghoos Y., Rutgeerts P. (1998). Digestibility of cooked and raw egg protein in humans as assessed by stable isotope techniques. J. Nutr..

[13] Bax M.L., Buffière C., Hafnaoui N., Gaudichon C., Savary-Auzeloux I., Dardevet D. (2013). Effects of meat cooking, and of ingested amount, on protein digestion speed and entry of residual proteins into the colon: a study in minipigs. PLoS One.

[14] Fuchs C.J., Hermans W.J., Smeets J.S., Senden J.M., van Kranenburg J., Gorissen S.H. (2022). Raw eggs to support postexercise recovery in healthy young men: did Rocky get it right or wrong?. J. Nutr..

[15] Nyakayiru J., van Lieshout G.A.A., Trommelen J., van Kranenburg J., Verdijk L.B., Bragt M.C.E. (2020). The glycation level of milk protein strongly modulates post-prandial lysine availability in humans. Br. J. Nutr..

[16] Chan A.H., D’Souza R.F., Beals J.W., Zeng N., Prodhan U., Fanning A.C. (2019). The degree of aminoacidemia after dairy protein ingestion does not modulate the postexercise anabolic response in young men: a randomized controlled trial. J. Nutr..

[17] Loveday S.M. (2023). Protein digestion and absorption: the influence of food processing. Nutr. Res. Rev..

[18] Trommelen J., Weijzen M.E.G., van Kranenburg J., Ganzevles R.A., Beelen M., Verdijk L.B. (2020). Casein protein processing strongly modulates post-prandial plasma amino acid responses in vivo in humans. Nutrients.

[19] Aguilera J.M., Lillford P.J., Watzke H., Aguilera J.M., Lillford P.J. (2008). Food Materials Science: Principles and Practice.

[20] Mosibo O.K., Ferrentino G., Alam M.R., Morozova K., Scampicchio M. (2022). Extrusion cooking of protein-based products: potentials and challenges. Crit. Rev. Food Sci. Nutr..

[21] Choton S., Gupta N., Bandral J.D., Anjum N., Choudary A. (2020). Extrusion technology and its application in food processing: a review. J. Pharm. Innov..

[22] Zhang J., Liu L., Jiang Y., Faisal S., Wang Q. (2020). A new insight into the high-moisture extrusion process of peanut protein: from the aspect of the orders and amount of energy input. J. Food Eng..

[23] Zhang J., Liu L., Liu H., Yoon A., Rizvi S.S.H., Wang Q. (2019). Changes in conformation and quality of vegetable protein during texturization process by extrusion. Crit. Rev. Food Sci. Nutr..

[24] Emin M., Teumer T., Schmitt W., Rädle M., Schuchmann H.P. (2016). Measurement of the true melt temperature in a twin-screw extrusion processing of starch based matrices via infrared sensor. J. Food Eng..

[25] Omosebi M.O., Osundahunsi O.F., Fagbemi T.N. (2018). Effect of extrusion on protein quality, antinutritional factors, and digestibility of complementary diet from quality protein maize and soybean protein concentrate. J. Food Biochem..

[26] Ghumman A., Kaur A., Singh N., Singh B. (2016). Effect of feed moisture and extrusion temperature on protein digestibility and extrusion behaviour of lentil and horsegram. LWT.

[27] Palanisamy M., Franke K., Berger R.G., Heinz V., Töpfl S. (2019). High moisture extrusion of lupin protein: influence of extrusion parameters on extruder responses and product properties. J. Sci. Food Agric..

[28] Onwulata C.I., Konstance R.P., Cooke P.H., Farrell H.M. (2003). Functionality of extrusion--texturized whey proteins. J. Dairy Sci..

[29] Rekola S.M., Kårlund A., Mikkonen S., Kolehmainen M., Pomponio L., Sozer N. (2023). Structure, texture and protein digestibility of high moisture extruded meat alternatives enriched with cereal brans. Appl. Food Res..

[30] Wang H., Wang R., Zhang L., Zhang W., Bakalis S., Li Y. (2023). Physicochemical properties, texture, and in vitro protein digestibility in high-moisture extrudate with different oil/water ratio. Food Res. Int..

[31] Day L., Swanson B.G. (2013). Functionality of protein-fortified extrudates. Compr. Rev. Food Sci. Food Saf..

[32] Lin S., Huff H.E., Hsieh F. (2002). Extrusion process parameters, sensory characteristics, and structural properties of a high moisture soy protein meat analog. J. Food Sci..

[33] Moughan P.J., Wolfe R.R. (2019). Determination of dietary amino acid digestibility in humans. J. Nutr..

[34] Trommelen J., M Holwerda A., Pinckaers P.J.M., van Loon L.J.C. (2021). Comprehensive assessment of post-prandial protein handling by the application of intrinsically labelled protein *in vivo* in human subjects. Proc. Nutr. Soc..

[35] Brodkorb A., Egger L., Alminger M., Alvito P., Assunção R., Ballance S. (2019). INFOGEST static in vitro simulation of gastrointestinal food digestion. Nat. Protoc..

[36] Tulbek M., Lam R.S.H., Wang Y.C., Asavajaru P., Lam A., Nadathur S.R., Wanasundara J.P.D., Scanlin L. (2017). Sustainable Protein Sources.

[37] Finnigan T.J.A., Phillips G.O., Williams P.A. (2011). Handbook of Food Proteins.

[38] Monteyne A.J., Coelho M.O.C., Porter C., Abdelrahman D.R., Jameson T.S.O., Jackman S.R. (2020). Mycoprotein ingestion stimulates protein synthesis rates to a greater extent than milk protein in rested and exercised skeletal muscle of healthy young men: a randomized controlled trial. Am. J. Clin. Nutr..

[39] Frayn K.N. (1983). Calculation of substrate oxidation rates in vivo from gaseous exchange. J. Appl. Physiol. Respir. Environ. Exerc. Physiol..

[40] Compher C., Frankenfield D., Keim N., Roth-Yousey L. (2006). Evidence Analysis Working Group, Best practice methods to apply to measurement of resting metabolic rate in adults: a systematic review. J. Am. Diet. Assoc..

[41] Flint A., Raben A., Blundell J.E., Astrup A. (2000). Reproducibility, power and validity of visual analogue scales in assessment of appetite sensations in single test meal studies. Int. J. Obes. Relat. Metab. Disord..

[42] Gallen I.W., Macdonald I.A. (1990). Effect of two methods of hand heating on body temperature, forearm blood flow, and deep venous oxygen saturation. Am. J. Physiol..

[43] Wolfe R.R., Chinkes D.L. (2004).

[44] Minekus M., Alminger M., Alvito P., Ballance S., Bohn T., Bourlieu C. (2014). A standardised static in vitro digestion method suitable for food–an international consensus. Food Funct.

[45] Smith P.K., Krohn R.I., Hermanson G.T., Mallia A.K., Gartner F.H., Provenzano M.D. (1985). Measurement of protein using bicinchoninic acid. Anal. Biochem..

[46] Khramtsov P., Kalashnikova T., Bochkova M., Kropaneva M., Timganova V., Zamorina S. (2021). Measuring the concentration of protein nanoparticles synthesized by desolvation method: comparison of Bradford assay, BCA assay, hydrolysis/UV spectroscopy and gravimetric analysis. Int. J. Pharm..

[47] Alam M., Kaur J., Khaira H., Gupta K. (2016). Extrusion and extruded products: changes in quality attributes as affected by extrusion process parameters: a review. Crit. Rev. Food Sci. Nutr..

[48] Horstman A.M.H., Ganzevles R.A., Kudla U., Kardinaal A.F.M., van den Borne J.J.G.C. (2021). Postprandial blood amino acid concentrations in older adults after consumption of dairy products: the role of the dairy matrix. Int. Dairy J..

[49] Paulussen K.J., Barnes T.M., Askow A.T., Salvador A.F., McKenna C.F., Scaroni S.E. (2023). Underpinning the food matrix regulation of postexercise myofibrillar protein synthesis by comparing salmon ingestion with the sum of its isolated nutrients in healthy young adults. J. Nutr..

[50] Fuller M.F., Tomé D. (2005). In vivo determination of amino acid bioavailability in humans and model animals. J. AOAC Int..

[51] Colosimo R., Warren F.J., Finnigan T.J.A., Wilde P.J. (2020). Protein bioaccessibility from mycoprotein hyphal structure: in vitro investigation of underlying mechanisms. Food Chem.

[52] Mession J.L., Sok N., Assifaoui A., Saurel R. (2013). Thermal denaturation of pea globulins (*Pisum sativum* L.)–molecular interactions leading to heat-induced protein aggregation. J. Agric. Food Chem..

[53] Sun X.D., Arntfield S.D. (2010). Gelation properties of salt-extracted pea protein induced by heat treatment. Food Res. Int..

