# Supplementary material for: High-Moisture Extrusion of a Dietary Protein Blend Impairs In Vitro Digestion and Delays In Vivo Postprandial Plasma Amino Acid Availability in Humans
Source: J Nutr. 2024 May 24;154(7):2053–64. doi: 10.1016/j.tjnut.2024.05.018 (PMC11282500; doi:10.1016/j.tjnut.2024.05.018)
Supplement: Multimedia component1 [file mmc1.docx]

**High moisture extrusion of a dietary protein blend impairs *in vitro* digestion and delays *in vivo* postprandial plasma amino acid availability in humans.**

**Supplementary materials**


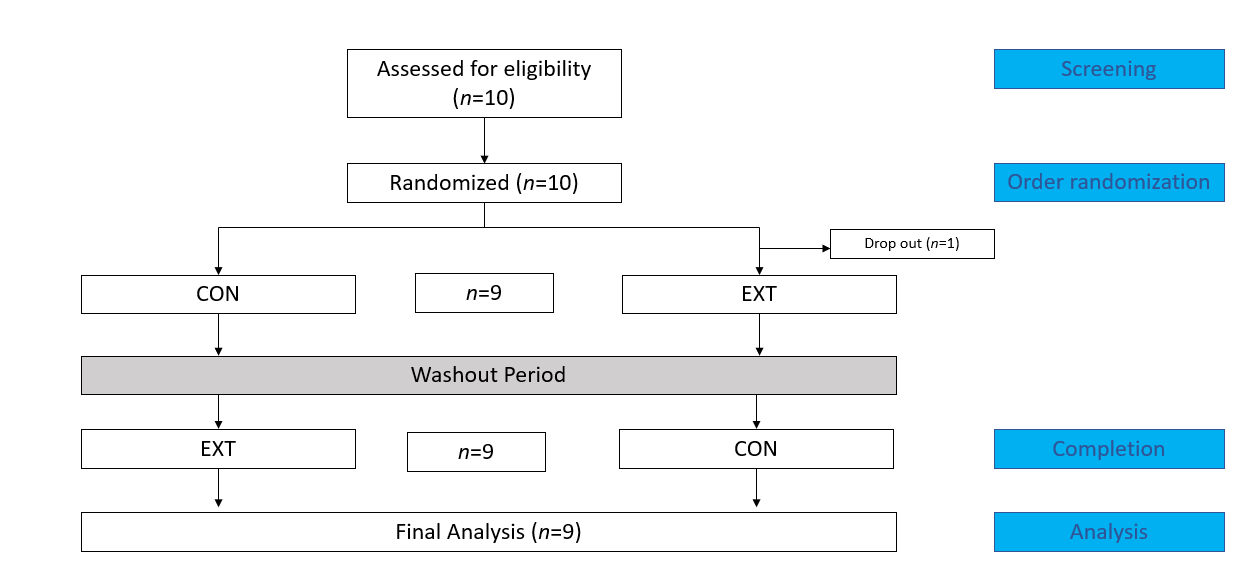


**Supplementary Figure 1.** Participant flow chart.

**
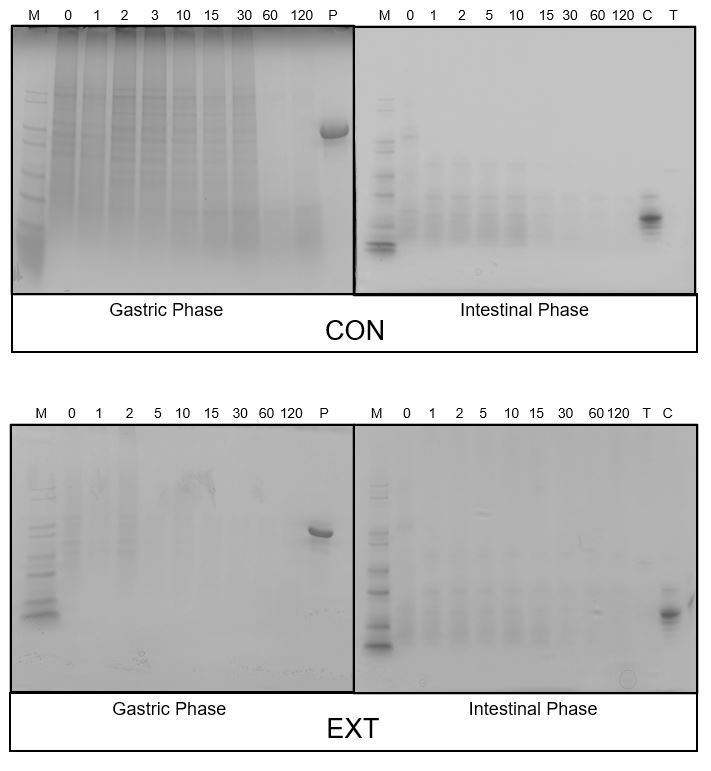
**

**Supplementary Figure 2.** SDS-PAGE protein hydrolysis for both CON and EXT conditions throughout INFOGEST static in vitro digestion (2 h gastric and 2 h intestinal phase). CON, dry blend; EXT, extrudate; M, protein molecular weight marker; P, pepsin; C, chymotrypsin; T, trypsin.
